# Beneficial insights into postbiotics against colorectal cancer

**DOI:** 10.3389/fnut.2023.1111872

**Published:** 2023-03-10

**Authors:** Dingka Song, Xiuli Wang, Yongjing Ma, Ning-Ning Liu, Hui Wang

**Affiliations:** State Key Laboratory of Oncogenes and Related Genes, Center for Single-Cell Omics, School of Public Health, Shanghai Jiao Tong University School of Medicine, Shanghai, China

**Keywords:** postbiotics, CRC, gut microbiota, nutritional intervention, immune regulation

## Abstract

Colorectal cancer (CRC) is one of the most prevalent and life-threatening cancer types with limited therapeutic options worldwide. Gut microbiota has been recognized as the pivotal determinant in maintaining gastrointestinal (GI) tract homeostasis, while dysbiosis of gut microbiota contributes to CRC development. Recently, the beneficial role of postbiotics, a new concept in describing microorganism derived substances, in CRC has been uncovered by various studies. However, a comprehensive characterization of the molecular identity, mechanism of action, or routes of administration of postbiotics, particularly their role in CRC, is still lacking. In this review, we outline the current state of research toward the beneficial effects of gut microbiota derived postbiotics against CRC, which will represent the key elements of future precision-medicine approaches in the development of novel therapeutic strategies targeting gut microbiota to improve treatment outcomes in CRC.

## 1. Introduction

As one of the most prevalent malignant tumors in the world, colorectal cancer (CRC) is a major cause of the steadily rising cancer death. According to a global statistical analysis covering 35 major types of cancer, CRC was ranked as the 3rd after female breast cancer and lung cancer, and 2nd most cancer-related deaths after lung cancer (1, 2). Currently, tumor resection surgery, radiotherapy, and chemotherapy have been widely used as the most commonly recommended treatment option in CRC (3). Targeted therapy has also emerged as another practical option of anti-cancer therapy in the past few decades (4). However, the therapeutic efficacy of current strategies is usually compromised in the late-stage or metastatic CRCs (5) while overall responsive in early-stage CRCs with a low level of relapse (6). It was reported that early diagnosis of CRC was accompanied with effective interventions which would largely increase the survival rate and overall wellness of patients (7). Therefore, accurate diagnosis and intervention at the early stage is necessary for CRC treatment.

During CRC development and progression, a large number of intrinsic and extrinsic risk factors have been identified (8). The hereditary non-polyposis colorectal cancer (HNPCC), well-known as Lynch syndrome, is attributed to autosomal dominant genetic mutations in one of four DNA mismatch repair (MMR) genes (9, 10). The environmental factors such as low physical activity, exposure to toxicities, etc., were also reported to facilitate CRC progression (11). Dietary patterns are considered as another critical factor in regulating gut microbiota homeostasis, which is detrimental for both initiation and progression of CRC (12–15). Inflammation, especially chronic inflammation, plays an important role in CRC development, as revealed by the fact that patients with inflammatory bowel disease (IBD) have higher risks of CRC ranging from 8.3 to 18.4% (16–18).

Recently, gut microbiota has been demonstrated to be closely associated with human health with the advancement of high-throughput multi-omics technologies. It was revealed that the abundance of gut microbes or their derived molecules are closely related to CRC, emphasizing their potential as biomarkers in early CRC diagnosis (19). Specifically, bacterial species like *Helicobacter pylori* were found to drive CRC development (20). *Fusobacterium nucleatum* has also been shown both positively associated with and contribute to the carcinogenesis of CRC (21, 22). *Porphyromonas* species were shown positively correlated with the progression of CRC, suggesting a causal effect of *Porphyromonas* on CRC tumorigenesis (23). In contrast, certain microbes may have functional roles in preventing CRC progression, as revealed by the protective effects through gut microbial manipulation (24). Additionally, probiotics as beneficial microorganisms for human, has been proven to improve the chemotherapy effectiveness of CRC (25). In spite of the benefits, precise manipulation of gut microbiota by dietary intervention or probiotic administration is still challenging nowadays due to many technical limitations. Of note, individual adhesion to nutritional plans varies that would markedly affect the efficacy of dietary interventions (26, 27). Furthermore, transplantation of live microbes into human brought additional concerns regarding the inevitable safety and ethical issues, which further dampens its clinical utilization (28).

Now a growing number of researches are turning to the alternative, postbiotics, which are defined by the International Scientific Association of Probiotics and Prebiotics (ISSAP) as a “preparation of inanimate microorganisms and/or their components that confers a health benefit on the host” (2). It should be noticed the term of “paraprobiotics” was used in previous studies to describe non-living microbial cells or cell fractions that confer health benefit to the host, in separating from the past definition of postbiotics as “soluble products or metabolites with beneficial functions” (29). To comply with the most recent guideline of postbiotics and also to give a full picture of recent advances in related fields, we chose to use the broader definition of postbiotics by the ISSAP in the present review.

**FIGURE 1:**
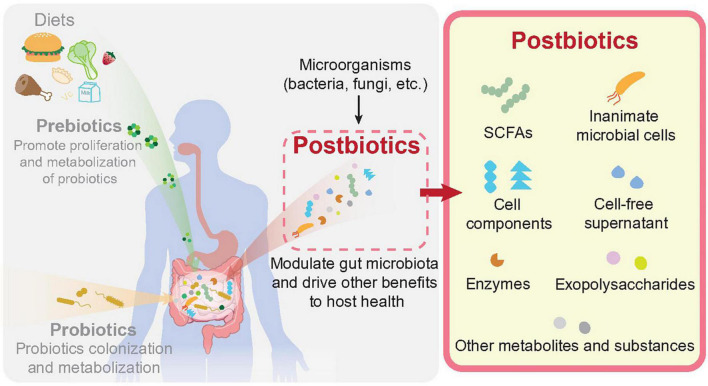
Prebiotics, probiotics, and postbiotics in human gut. As described in the main text, prebiotics are produced through consumption of daily intakes by our digest system, which are selectively utilized by host microorganisms conferring health benefits. Probiotics are defined as live microorganisms residing in human body, notably the intestinal lumen, which are beneficial for human health. Postbiotics are preparations of microbial derived substances that contribute to the health of host. Postbiotics can be derived from variable microbial origins, including cell components, cell-free supernatants, cell wall structures like exopolysaccharides (EPS), metabolites like short chain fatty acids as well as enzymes etc.

Distinct from the “prebiotics” defined as “a substrate that is selectively utilized by host microorganisms conferring a health benefit” (30), or the “probiotics” defined as “live microorganisms that, when administered in adequate amounts, confer a health benefit on the host” (31), the importance of postbiotics in regulating gut homeostasis remains at its infancy stage. As postbiotics are beneficial substances derived from microorganisms, they must be delivered at host surfaces through various routes to achieve their biological effects, such as oral cavity, gut, skin, urogenital tract, nasopharynx etc., (32). Previous studies have already uncovered the biological activities of gut microbe-derived metabolites in maintaining overall health and protecting against various diseases (33–35). In the context of CRC, the benefits of postbiotics have also been uncovered (36). Compared with live microorganisms (probiotics for instance), the molecular mechanisms of postbiotics as well as their biological effect are easier to be evaluated (28). A brief illustration of prebiotics, probiotics and postbiotics in the context of gut is displayed in Figure 1. Upon diet consumption, prebiotics can be released for gut microbial utilization. Outgrowth of gut commensals (or probiotics in the context of gut tissue), can exert multiple benefits on human health. Postbiotics, which are derived from both probiotics or many other kinds of microorganisms, can be naturally produced or directly applied on host surfaces for the improvement of human gut health and CRC prevention/treatment.

Although it is clear that the microbial sources or “sites of actions” of postbiotics are not limited to gut, the well-established relationship between gut microbiota and CRC make it a valuable topic to investigate the potential effects of gut microbial derived postbiotics in CRC progression. Here, we reviewed the current advances regarding the role of gut microbial derived postbiotics in CRC, including the identification, functional mechanism, and potential clinical applications.

## 2. Identification of postbiotics from multi-kingdom gut microbiota

Postbiotics are composed of various types of microbial preparations including microbial cells but also various peptides, vitamins, and structural components, etc., (37). Although it was proposed that chemically identified molecules from microorganisms were no longer suitable to be named as postbiotics (2), we prefer to include those molecules in this retrospective review as they were isolated from microorganisms and demonstrated to be effective against CRC, which would be insightful for future studies in related field.

Gut microbiota inhabited in the human gastrointestinal tract along with their derived metabolites are considered to be detrimental in human health and diseases (37). Growing evidence has demonstrated the close relationship between the abundance or activity of certain microbes and the incidence of various diseases. The importance of non-living microbial cells and their metabolites has also been recognized recently (34). Previous studies identified bioactive ingredients from gut microbiota by traditional biochemical approaches (centrifugation, ultrafiltration, metabolomics, etc.) (38). However, the biological functions of postbiotics in human health particularly the role of postbiotics in CRC initiation, progression, as well as treatment, are still poorly understood. Here described different types of postbiotics uncovered from bacteria, fungi as well as other microorganisms residing in the gut. The specific role of each type of postbiotics as well as the underlying mechanisms are illustrated in later sections. A concise list of different types of identified postbiotics, their source of identification, biological effects in CRC as well as the potential mechanisms are also summarized (Table 1).

**TABLE 1:** List of identified postbiotics and beneficial effects for colorectal cancer (CRC) treatment.

Types	Sources	Examples	Effects on CRC	Potential mechanisms	References
Heat-killed microorganism cells	Bacteria Fungi	heat-killed *Bifidobacterium breve* MG731 heat-killed *Lactobacillus reuteri* MG5346 heat-killed *Lactobacillus casei* MG4584 heat-killed yeast cells	Maintain intestinal homeostasis Anti-inflammatory activities Induce apoptosis of human colorectal carcinoma RKO cell	Activate caspase-9-dependent apoptosis	(51, 57)
Cell-free supernatant	Bacteria Fungi	CFS of *Lactobacillus* species CFS of *Bifidobacterium* species CFS of *Lacticaseibacillus* species CFS of *Saccharomyces cerevisiae*	Antimicrobial/Antioxidant/Antitumor activity. Induce apoptosis of colorectal cancer cells *in vitro*	Unclear	(16, 58–63)
Cell component	Bacteria	wall teichoic acids (WTAs); lipoteichoic acids (LTAs);	Modulation of gut microbiota; Anti-inflammatory activities;	Modulate TLR-2/p38-MAPK/NF-kB pathway	(29, 68–71)
		Peptidoglycan (PGN)	Induce autophagy and apoptosis	Modulate Nod1/CARD/RIP2/NF-kB/MAPK pathway	(78)
	Fungi	Yeast cell wall (YCW)	Maintain intestinal homeostasis; Anti-inflammatory activities; Antiproliferative activity	Unclear	(145–147, 149–152)
SCFA	Bacteria	Butyrate acid; Acetate acid; Propionate acid; Valerate acid	Inhibit the proliferation of colon cancer cells Increase proliferation of colon crypts Anti-inflammatory activities	Interact with GPCR41/43 and GPCR109a Downregulation of ERK/MAPK signals Upregulation of Camp/PKA/CREB/HDAC signal and WNT signals	(100, 258, 259, 264)
EPS	Bacteria Fungi	HoPS HePS PSA Mannan-oligosaccharides	Induce apoptosis of colon cancer cells Induce G0/G1 cell cycle arrest of colon cancer cells Prevents the proliferation and colonization of bacterial pathogens	Interact with innate immune sensing receptors including TLR2 Regulate innate immune responses Suppress IL-17 production	(80, 84, 85, 153, 274)
Enzyme	Bacteria Fungi Archaea	β-glucuronidase; nitro-reductase; mucinase; catalase; superoxide dismutase; glycosyltransferases	Suppress chemicals (AOM, DMH, TNBS) induced colon cancer in animal models	Unclear	(107, 109, 285)
Bile acids	Bacteria	deoxycholate acid	Induce apoptosis of colon cancer cells	Unclear	(294)
Tryptophan metabolites	Bacteria	Indole metabolites Kynurenine Serotonin	Boost clearance of cancer cells by host immune system Alleviate colitis	Interact with aryl hydrocarbon receptor Regulate colon barrier function Induce Treg differentiation. Confine Th17 and Th1 responses Produce anti-inflammatory mediators	(299, 300, 302)
Bacteriocins	Bacteria	Salivaricin Plantaricin JLA-9/W Lactococcin A/mmfii;	Antimicrobial activity Anti-inflammatory activities. Induce autophagy and apoptosis	Modulate MAPK/NF-κB/COX-2 pathway Modulate PI3K/AKT/caspase 3 apoptosis pathway Modulate PI3K/AKT/Atg 4/5/7/3/12 autophagy pathway	(139, 140)
Vitamins	Bacteria	B-group vitamins Vitamin K	Unclear	Unclear	(141, 142)
Neurotransmitter substance	Bacteria	γ-aminobutyric acid 5-hydroxytryptamine; dopamine; norepinephrine Brain-derived neurotrophic factor Acetyl choline	Unclear	Unclear	(29, 143, 144)

This table summarized various types of currently identified postbiotics, their microbial sources, the effects on CRC as well as the underlying mechanism. CFS, cell free supernatant; WTA, wall teichoic acids; LTA, lipoteichoic acids; PGN, peptidoglycan; YCW, Yeast cell wall; HoPS, homopolysaccharide; HePS, heteropolysaccharide; SCFA, short chain fatty acids; EPS, exopolysaccharides; PSA, polysaccharide; AOM, Azoxymethane; DMH, 1,2-dimethyl hydrazine; TNBS, 2,4,6-Trinitrobenzenesulfonic acid; TLR, toll like receptor; MAPK, mitogen-activated protein kinase; NF-kB, nuclear factor kappa-light-chain-enhancer of activated B cells; Nod1, nucleotide-binding oligomerization domain-containing protein 1; CARD, caspase activation and recruitment domains; RIP2, receptor-interacting serine/threonine-protein kinase 2; GPCR, G protein-coupled receptors; ERK, extracellular signal-regulated kinase; PKA, protein kinase A; CREB, cAMP response element-binding protein; HDAC, histone deacetylases; IL, Interleukin; Th, T helper.

### 2.1. Postbiotics from gut bacteria

#### 2.1.1. Heat-killed bacterial cells

The heat-killed microbial cells had not been included into postbiotics until 2021, when the International Scientific Association of Probiotics and Prebiotics (ISAPP) incorporated the deliberately inanimate microbial cells, mostly heat-killed probiotic bacteria cells, into the definition of postbiotics (2, 39–41). To address the biological role of microbial cells, microorganisms were further inanimate by various methods like heating, chemical (e.g., formalin), gamma or ultraviolet rays, and sonication treatments (42–44). *Lactobacillus* and *Bifidobacterium* species, the mostly studied probiotics, were found to have beneficial functions in heat-killed formations (45–51). For instance, heat-killed *Lactobacillus salivarius* subsp. *salicinius* AP-32, *L. rhamnosus* CT-53, *L. paracasei* ET-66 could significantly inhibit the invasion of oral pathogens to improve oral health (45). Besides, heat-killed *Lactobacillus* and *Bifidobacterium* species are found to exhibit immunomodulatory effect. Heat-killed *Bifidobacterium breve* M-16V cells suppressed proinflammatory cytokine production in spleen cells and affected intestinal metabolism (46). Oral administration of heat-killed *L*. *plantarum* L-137 could enhance protection against IFV infection together with increased IFN-β production in the serum of infected mice at an early stage of infection (47). *Lactobacillus rhamnosus* GG (LGG) could ameliorate intestinal inflammation, and promote cytoprotective responses in the developing murine gut, thus playing a role in inflammatory bowel disease treatment (16, 48, 49). Li et al. found the heat-killed LGG could also decrease lipopolysaccharide (LPS)-induced proinflammatory mediators and increase anti-inflammatory mediators (50). Ueno et al. proved that administration of heat-killed *Lactobacillus brevis* SBC8803 could successfully maintain intestinal homeostasis and cure intestinal inflammation, which manifesting the potential for CRC treatment (51). Recently, *Akkermansia muciniphila* was found to be beneficial in the prevention of various diseases through maintaining the integrity of gut barrier (52). Implantation of live *Akkermansia muciniphila* exerted protective role against liver injury, colitis and CRC as well (53–55). Interestingly, Wang et al. showed that the pasteurized bacterium of *Akkermansia muciniphila* strikingly inhibit colitis associated tumorigenesis in mice, indicating the beneficial role of *Akkermansia muciniphila* derived components against CRC progression (56). Meanwhile, Kim et al. reported that heat-killed *B. bifidum* MG731, *L. reuteri* MG5346, and *L. casei* MG4584 significantly delayed tumor growth in colorectal carcinoma, which give direct evidence of heat-killed bacterial cells for CRC (57).

#### 2.1.2. Cell-free supernatant (CFS)

Cell-free supernatant refers to the biologically active metabolites secreted by microorganisms. Here we mainly focus on the cell-free supernatant of bacteria, which are prepared after the bacterial cells were incubated, centrifuged, and removed (34). CFS has been reported to show antimicrobial, antioxidant, and antitumor activity (16, 58–63).

The antimicrobial activity refers to both antibacterial and antifungal activity (58, 59). The *Saccharomyces cerevisiae* CFS showed anti-biofilm activity against *Staphylococcus aureus*, which is the widely reported pathogen in both food and clinical environment (58, 64). The anti-biofilm property of *S. cerevisiae* CFS can be thus utilized to reduce the risk of infection by *S. aureus* in human (65). In addition, the cell-free supernatants derived from *Lactobacillus paracasei* subsp. *Paracasei* SM20 and *Propionibacterium jensenii* SM11 coculture all showed high antifungal activity against *Candida pulcherrim* and *Rhodotorula mucilaginosa* (59). Interestingly, CFS of *B. animalis subsp. Lactis* DSMZ 23032, *L. acidophilus* DSMZ 23033, *L. rhamnosus* SD11, and *Lactobacillus brevis* DSMZ 23034 exhibited strong antioxidant activity through DPPH radical scavenging and oxidative stress hypersensitivity *in vivo* (60). Pourramezan et al. found that there was a relationship between the antibacterial activity and antioxidant activity of CFS of *Lactobacillus* (61).

For *Lactobacillus* and *Bifidobacterium* species, their CFS were found to inhibit cancer progression (62). For example, *L. casei* and LGG cultures could prevent the invasion of colon cancer cells (62). In recent years, *Lactobacillus* cell-free supernatant (LCFS) has attracted more and more attention to its potential benefits on CRC prevention (63). The anti-cancer effects of *L. fermentum* CFS has been tested which proved that it could induce apoptotic cell death in three dimensional (3D) spheroids of colorectal cancer cells *in vitro* (63). Besides, the mRNA levels of apoptosis markers were dramatically induced after treating with LCFS in 3D system (63). Pahumunto et al. showed that CFS of *Lacticaseibacillus paracasei* SD1, *Lacticaseibacillus rhamnosus* SD4, *Lacticaseibacillus rhamnosus* SD11, and *Lacticaseibacillus rhamnosus* GG could inhibit pro-inflammatory cytokine expression in Caco-2 cells and inhibit the growth of Caco-2 cells, supporting its role in preventing CRC (16).

#### 2.1.3. Cell components

According to the more recent definition of postbiotics by the ISSAP, cell components can be regarded as another major type of postbiotics. Two categories of cellular components were intensively investigated by far. Teichoic acids (TAs) are major constituent of bacterial cell walls, including both wall teichoic acids (WTAs) covalently linked to peptidoglycan and lipoteichoic acids (LTAs) anchored to the cytoplasmic membrane (66, 67). WTA was reported to be effective in modulating bacterial colonization (29), while LTA was discovered to exert immune-modulatory activity by recognizing Toll-like receptors (68). LTA could interact with TLR2 and TLR6 to active host immune response (69). In particular, LTA of *Lactobacillus paracasei* D3-5 could enhance mucin (Muc2) expression by modulating TLR-2/p38-MAPK/NF-kB pathway, which in turn reduces inflammation (70). In addition, LTA of *Lactobacillus plantarum* inhibited the phosphorylation of ERK and p38 kinase as well as the activation of NF-κB, resulting in decreased IL-8 production and inflammation suppression in porcine intestinal epithelial cells (71).

Peptidoglycan (PGN) is the conserved structure of Gram-negative bacterial which could be recognized by nucleotide-binding oligomerization domain-1 (NOD1) (72). Once activated by PGN, NOD1 could further activate the downstream NF-κB (72) and MAPK (73) pathway through interacting with a caspase activated and recruitment domain (CARD) and its adaptor protein, the receptor-interacting protein 2 (RIP2) (74–76). Besides, the PGN/Nod1/RIP2 pathway could induce autophagy and inflammatory signaling in response to bacterial infection (77). PGN of the *Lactobacillus paracasei* subsp. *paracasei* M5 strain exerted cytotoxic effects on HT-29 cells and induced a mitochondria-mediated apoptosis in colon cancer (78).

#### 2.1.4. Exopolysaccharides (EPS)

Exopolysaccharides (EPS) is a subclass of polysaccharides that constitute the cell wall structure of bacteria and is characterized by less attachment to the cell surface compared with capsular polysaccharides (79). EPS can be structurally divided into two categories: homopolysaccharide (HoPS) and heteropolysaccharide (HePS). HoPS was composed of a single type of monosaccharides, such as *D*-glucose or *D*-fructose. Crosslinking of *D*-glucose through α- or β-glycosidic linkage resulted in α- or β- glucans, respectively, while connection of *D*-fructose through β-glycosidic linkage forms β-fructans (80). In contrast, HePS are composed of different types of monosaccharides, such as *D*-glucose, *D*-galactose, and *L*-Rhamnose (80). Besides, *N*-acetylation is also found to be presented on HePS, which further diversified their molecular structures (81).

Bacterial EPS has been evaluated as industrially important biopolymers with significant commercial potential (82). The effects of EPS on human health (and in particular, CRC) have also been recognized (80). EPS can be synthesized by a wide range of bacterial species such as *Bifidobacteria* sp. and *Lactobacillus* sp., which benefits the gut homeostasis (83). The protective role of EPS in CRC has been demonstrated previously. The EPS extracted from *Lactobacillus acidophilus* was found to inhibit the growth of Caco-2 cells in a dose-dependent manner (84). Similarly, *Lactobacillus*-derived EPS was found to suppress HT-29 cell growth by inducing the G0/G1 cell cycle arrest and apoptosis (85). In line with that, EPS produced by microalgae *Chlorella pyrenoidosa*, *Scenedesmus* sp., and *Chlorococcum* sp. was found to induce anti-tumor effect on both HCT116 and HCT8 cells (86), similar to EPS produced from *Chlorella zofingiensis* and *Chlorella vulgaris* (87). Taken together, these data suggest a universal protective effect of EPS against CRC independent of its sources. Nevertheless, the *in vivo* activity of EPS in CRC protection has not been fully clarified yet. It was revealed that EPS from *Lactobacillus rhamnosus* is critical in providing the “shield” for bacteria itself against the host immune defense, which is beneficial for the maintenance of gut microbiota homeostasis (88). Apart from that, EPS from *Bifidobacterium longum subsp. Longum s*ignificantly alleviated DSS-induced colitis by maintaining the integrity of gut mucosal barrier (89), which was supported by the significantly altered level of EPS from *Rhizopus nigricans* in CRC mouse model (90). Taken together, these studies support the beneficial role of EPS against CRC.

#### 2.1.5. Short chain fatty acids (SCFAs)

Short chain fatty acids (SCFAs) are a subset of saturated fatty acids with six or less carbon molecules including acetate, propionate, butyrate, pentatonic (valeric) acid, and hexanoic (caproic) acid (91). SCFAs are the main metabolic products of indigestible saccharides by anaerobic bacterial fermentation in human gut (91). Deficiency of SCFAs production can lead to the pathogenesis of many human diseases, such as autoimmune syndromes from allergies to asthma, metabolic diseases, neurological diseases, and cancers (92).

The protective effects of SCFAs against colorectal cancer were first discovered in the early 1980s. Whitehead et al. found that butyrate stimulation significantly inhibited the proliferation rate of human colorectal cancer cell line LIM1215 (93), accompanied by a similar finding that both propionate and butyrate suppressed the growth of human colorectal cancer HT-29 cells (94). In an independent study, it was shown that SCFA treatment, either alone or in combination, significantly increased the proliferation of colon crypts, suggesting a symbiotic effect on colorectal health, which eventually prevents the formation or progression of colorectal cancer (95). The anti-cancer effect of SCFAs is also supported by epidemiological studies revealing an inverse correlation between the level of dietary fibers and the incidence of human colorectal cancer (96). In line with this, the proportions of major SCFAs in enema samples of patients with colon polyps or colorectal cancer were strikingly different from healthy subjects, indicating the niche-dependent activity of SCFAs in response to CRC (97). Moreover, Gibson et al. investigated the fermentative production of butyrate *in vivo* as well as its protective effect against colorectal cancer in a rat model (98). It was found that the intake of natural fiber was associated with high concentration of butyrate in colon tissue and reduction of colorectal tumor formation, which highlighted the protective effect of butyrate in colorectal cancer. Propionate and valerate are also members of SCFAs effective in arresting cell growth or suppressing differentiation of human colon carcinoma cells (99). Although the *in vivo* activity of SCFAs in the context of CRC is still under investigation, the anti-inflammatory effects of SCFAs have been well-described and are essential for the inhibition of colitis and inflammatory bowel disease (IBD), which are both considered as the major risks for the development of CRC (100, 101). Of note, recent studies uncovered the physiological role of SCFA in preventing colitis-induced CRC in mice, as administration of SCFA mix strongly suppressed tumor burden in mice treated with Azoxymethane (AOM)/dextran sodium sulfate (DSS) which were commonly used for the induction of colitis-associated colorectal cancer (100). Colon inflammation as well as pathological score index was also reduced, consistent with the decreased secretion of pro-inflammatory cytokines including interlukin-17 (IL-17), IL-6, and tumor necrosis factor-α (TNF-α) (100, 102). Overall, these results highlighted the clinical potential of SCFAs in protection against CRC.

#### 2.1.6. Enzyme

Enzymes are recognized as key regulators driving the metabolism of all organisms, with no exception for gut microbiome (103). In fact, enzymes encoded by microbes play essential roles in host-microbe interactions (103). Early studies on the epidemiology of CRC revealed that there was a negative correlation between the abundance of lactic acid-producing bacteria *Bifidobacterium* in human gut and the incidence of colorectal cancer (104). Lactic acid bacteria (LAB) are active in fermentation *via* synthesizing various enzymes, and the protective effects on colorectal cancer by dietary supplementation of different lactic acid-producing bacterial species have been well-established in murine studies (105–108). Therefore, the potential role of bacterial enzymes in inhibiting CRC development has also been indicated. In fact, it was shown that administration of *Bifidobacterium longum* and *Lactobacillus acidophilus* generated better outcome of AOM-induced colorectal cancer in rats, and was accompanied by increased β-glucuronidase and nitro-reductase activity in fecal samples (109). It was also demonstrated that the inhibitory effect of *Bifidobacterium longum* on colon carcinogenesis was induced by 2-Amino-3-methylimidazo [4,5-f] quinoline (IQ), a food mutagen (106). Consistently, supplementation of coconut cake was found to remarkably reduce the incidence of 1,2-dimethyl hydrazine (DMH)-induced colorectal cancer occurrence in a rat model, which was associated with the upregulation of β-glucuronidase and mucinase (107). In addition, oral administration of a catalase-producing *Lactococcus lactis* can prevent DMH-induced colorectal cancer in mice (108).

#### 2.1.7. Other bacterial derived components

Other types of metabolites produced by gut microbes are also involved in regulating CRC progression, which include the microbially modified secondary bile acids (secondary BAs). Primary bile acids (primary BAs) are cholesterol derivatives that synthesized in the liver and then secreted into intestine for lipid absorption. Once utilized, most bile acids are recycled to liver *via* enterohepatic or systemic circulation. Meanwhile, a proportion of primary BAs is transported to colon where they are metabolized by gut microbiome to produce secondary BAs. The detailed signal transduction pathways of BA metabolism have been comprehensively reviewed (110). The relationship of diet, BAs, gut microbiota and CRC has been revealed in recent studies (110, 111). It was reported that the consumption of “unhealthy” western diet is associated with the increase of secondary BAs, especially deoxycholic acid (DCA) and lithocholic acid (LCA) in the gut, while the increased level of bile acids is tightly correlated with high CRC incidence (112–115). Therefore, secondary BAs are primarily considered as carcinogens in human gastrointestinal cancers (116). In addition, high-fat diets promote the synthesis and colonic delivery of bile acids, which stimulate the growth and activity of 7α-dehydroxylating bacteria to convert the primary BAs into secondary BAs (117). Animal studies also showed the effects of high fact diet in modulating the composition and activity of gut microbiota, which lead to increased BA secretion and colon tumorigenesis (118–120). Diet intervention has been implicated to be critical in preventing CRC through the regulation of BA levels in gut (121). These results indicate the biological role of gut microbial secreted BA in regulating the development of CRC. On the other side, ursodeoxycholic acid (UDCA), another member of secondary BAs (122), was effective in inducing apoptosis of colon cells *in vitro* (123), which is distinct from DCA (124). Consistently, another study uncovered the role of UDCA in inhibiting the proliferation of colon cancer cells by regulating oxidative stress and cancer stem-like cell growth (125). Mechanistically, it was revealed that UDCA suppressed the malignant progression of colorectal cancer by inhibiting the activation of Hippo/YAP pathway (126). Animal studies demonstrated the protective role of UDCA in chemical induced colon tumorigenesis (127, 128). According to a cross-sectional study, by analyzing the relationship between ursodiol use and colonic dysplasia (the precursor to colon cancer) in patients with ulcerative colitis and primary sclerosing cholangitis, it was revealed that the application of UCDA is associated with lower prevalence of colonic neoplasia (129). Although UDCA has been approved for the treatment of primary biliary cirrhosis by FDA (130), the mechanism of action of UCDA, the relationship of UCDA with gut microbiota, as well as the clinical use of UCDA in CRC treatment remains undetermined and therefore warrants further investigation.

Tryptophan is one of the essential amino acids supplied by the intake of dietary proteins (131). Despite the absorption of the majority of ingested proteins in small intestine, a small fraction of amino acids can reach colon where they can be absorbed by local microbes (132). Tryptophan metabolism is complicated and dependent on two different pathways: the kynurenine (Kyn) pathway and the indolic pathway (133). The significance of microbial tryptophan metabolites in human health has been investigated (134). In the context of CRC, it has been shown that Tryptophan metabolites derived from gut microbes regulate the homeostasis of gut immunity. The deficiency of Tryptophan secretion due to gut dysbiosis may cause exacerbated inflammatory response which eventually led to colorectal tumorigenesis (135). In fact, alteration of fecal tryptophan metabolism was correlated with shifted microbiota, which may be involved in the pathogenesis of colorectal cancer (136).

Bacteriocins are defined as ribosomally synthesized antimicrobial proteins or peptides. It could be divided into two categories: class I bacteriocins are modified by post-translational modifications; class II bacteriocins remain unaltered (137, 138). Given the fact that bacteriocins have antimicrobial activity, bacteriocins targeting CRC associated bacterial pathogens may have potential benefits against CRC. For example, *Streptococcus salivarius* DPC6993 which could secrete salivaricin against *F. nucleatum* in an *ex vivo* model of human colon, was shown to be effective in reducing the risk of CRC (139). Probiotics-derived bacteriocins, including plantaricin JLA-9, plantaricin W, lactococcin A, and lactococcin MMFII directly interact with CRC-promoting protein COX2 and modulate inflammasome complex NLRP3 and NF-kB pathways to reduce CRC-associated inflammation. They also have the potential of activating autophagy and apoptosis by regulating PI3K/AKT and caspase pathways in CRC (140).

In addition to the above-mentioned molecules, several other types of gut microbial derived molecules were also beneficial to human health. For instance, vitamins secreted by gut microbes, including B-group vitamins and vitamin K, were shown to be effective in preventing CRC (141, 142). Neurotransmitter substances synthesized by gut microbes, such as γ-aminobutyric acid (GABA), 5-hydroxytryptamine (5-HT), dopamine (DA), norepinephrine (NE), brain-derived neurotrophic factor (BDNF), and acetyl choline (29), were critical in regulating gut-brain axis (143, 144). However, their roles in CRC still awaits further investigation. In conclusion, a variety of probiotic bacteria-derived molecules have been identified, and the potential of these bacterial derived “postbiotics” in preventing/treating CRC have also been highlighted.

### 2.2. Postbiotics from gut fungi

#### 2.2.1. Yeast cell wall (YCW) structural components

Yeast is one kind of highly adaptable microorganisms with proved benefits to human health. Yeast cell wall, particularly the mannan-oligosaccharides, were reported to be able to bind to fimbriated bacteria in the gut and prevent their attachment to gut mucosa which eventually prevents the proliferation and colonization of those bacterial pathogens (145). Furthermore, mannan-oligosaccharides (MOS) in the YCW have a strong binding affinity with type I-fimbriae in the gut (146, 147). The probiotic yeast *S. boulardii*, widely incorporated in dairy food, grains, fruit juices, chocolate, coffee, and tea, was demonstrated to ameliorate diarrheal conditions. Cell wall extracts of *S. cerevisiae* and *S. boulardii* could adsorb aflatoxin B1 (148), cholera toxin (149), and pathogenic *E. coli* (150) which are possibly by cell wall polysaccharides such as β-glucan, mannoprotein and chitin (148, 151). Furthermore, by adsorbing these, the cell wall extracts of *S. boulardii* could enhance immune responses in the intestinal mucosa (152). As for the relationship between YCW and CRC, the β-glucan of *S. boulardii* cell wall extract has been proven to prevent colon carcinogenesis *in vitro* (151). In addition, EPS was also found in probiotic yeast species like *Kluyveromyces marxianus* and *Pichia kudriavzevii*, indicating the complicated natural source of EPS (153). Similarly, EPS derived from yeast species *Kluyveromyces marxianus* and *Pichia kudriavzevii* showed a comparable anti-colorectal cancer activity (153). Collectively, these results highlight the potential of YCW as postbiotics against CRC.

#### 2.2.2. Other compounds of fungi

The anaerobic gut fungal commensals (such as *Anaeromyces robustus*, *Caecomyces churrovis*, *Neocallimastix californiae*, and *Piromyces finnis*) are reported to encode diverse biosynthetic enzymes for natural products and antimicrobial peptides (AMP), including polyketide synthases (PKS), non-ribosomal peptide synthases (NRPS), etc., (154). A significant proportion of fungal products were detected in the fecal samples of gnotobiotic mice, which may affect the growth and differentiation of host epithelial and immune cells (155).

A postbiotic yeast cell wall-based blend containing hydrolyzed yeast cell wall of *Saccharomyces cerevisiae*, organic acids (n-butyric acid), vitamins (ascorbic acid), and essential oils can partially improve the health of pigs which were treated with multiple mycotoxins including aflatoxin B1 and deoxynivalenol (156). The potential of using *S. boulardii*-derived postbiotics (for instance, polyamines, organic acids, enzymes, etc.) as dietary supplements has also been suggested (157). However, the benefits of non-YCW fungal products in CRC remain unclear which awaits further investigation in the future.

### 2.3. Postbiotics from non-bacterial/fungal gut microbes

Methanogenic archaea are the most well-studied archaea in human gut. While archaea do not produce enzymes to break down complex carbohydrates for nutrition (which may rely on other symbiotic species in the gut), they do have special glycosyltransferases that can link monosaccharides to various ligands to form glycoconjugates (158). Furthermore, it was indicated that methanogens can downregulate gut trimethylamine (TMA) concentration through specific metabolism of trimethylamine depletion (159, 160), highlighting the probiotic effect of methanogens on human health. However, it remains largely unknown about the involvement of fungus or archaea-derived postbiotics in CRC progression/inhibition, which relies on comprehensive studies in the future.

### 2.4. Inter-kingdom interaction between postbiotics and CRC

Although the biological functions of individual postbiotics have been comprehensively investigated, the interactions between postbiotics derived from multi-kingdom gut microbiota as well as their roles in the development of CRC were also worthy to be evaluated. Coker et al. showed the ecological network between bacteria and fungi in healthy gut, which is disrupted in CRC patients, and the antagonized interkingdom interactions contribute to the progression of CRC (161). In another study, the interaction of bacteria and archaea has been shown important for CRC development, as healthy individuals have a positive association between bacterial and archaeal diversity, which disappeared in patients with CRC (162). Furthermore, Liu et al. recently investigated the four-kingdom microbiota alterations with CRC metagenomic datasets from 8 distinct geographical cohorts and finally demonstrated the feasibility of multi-kingdom markers as CRC diagnostic tools (163). Narunsky-Haziza et al. explored the mycobiome in 35 cancer types including colorectal cancer from 4 international cohorts. They discovered the widely fungal existence and the permissive co-occurring fungi-bacterial ecologies across diverse cancer types. Thus, prognostic and diagnostic capacities of the tissue and plasma mycobiomes together with bacteriomes were suggested (164). The role of inter-kingdom interactions of microorganisms in CRC has been uncovered, however, whether it affects the inter-kingdoms interaction of postbiotics and CRC progression still need to be investigated in the future.

## 3. Biological functions of postbiotics against colorectal cancer

### 3.1. *In vitro* biological activities of postbiotics

#### 3.1.1. Inhibition of cancer cell proliferation

The current hallmarks of cancer embody eight hallmark capabilities and two enabling characteristics, include sustaining proliferative signaling, evading growth suppressors, avoiding immune destruction, enabling replicative immortality, tumor-promoting inflammation, activating invasion and metastasis, inducing or accessing vasculature, genome instability and mutation, resisting cell death and deregulating cellular metabolism (165, 166). With the growing evidence and our increasing understanding of cancer biology, emerging hallmarks and enabling characteristics are now introduced into the hallmarks of cancer, involving “unlocking phenotypic plasticity,” “non-mutational epigenetic reprogramming,” “polymorphic microbiomes,” and “senescent cells” (167). Among these, excessive cell proliferation mainly resulted from three types of mutations: mitogen or its signal transducing molecules to convey mitogen information, mutation of late-G1 cell-cycle checkpoints regulated by pRB, and uncontrolled expression of Myc (168–170). Therefore, one of the anti-cancer strategies is to block cancer cell proliferation, and that is how most postbiotics works against cancer (171). It was observed that the ratio of G0/G1 cells increased when treated with gemcitabine together with butyrate, a common postbiotic from intestinal bacteria. The percentage of cells in S-phase significantly decreased when treated with butyrate in PANC-1 cell, indicating that butyrate could inhibit the proliferation of human pancreatic cancer cells (172). Postbiotic metabolites produced by certain strains of *L. plantarum* exhibited selective cytotoxic activity *via* antiproliferation of human breast cancer cells, suggesting their potential against breast cancers (173). In addition, Lazarova et al. proposed that butyrate can induce cell cycle arrest which was dependent on the modulation of Wnt signal activity in CRC (174). When combined with chemotherapeutic agents like irinotecan, butyrate significantly inhibited cell proliferation which promoted the efficacy of chemotherapies for the treatment of colorectal cancer (175).

In addition to butyrate, varies of postbiotics derived from *Lactobacillus* also exhibited anti-proliferative activities on colorectal cancer cells. For instance, the cell-free culture supernatant of *Lactobacillus* has inhibitory effect on the proliferation of colorectal cancer cells (176). Another interesting study reported that cell free cultural supernatants of *Lactobacillus* conjugated with various linoleic acids significantly reduced the transcriptional level of genes required for colon cancer cell growth and proliferation, such as CDK1/2/6, PLK1, and SKP2, indicating the potential benefits of those metabolites in treating CRC (177).

#### 3.1.2. Induction of cancer cell apoptosis

Cell apoptosis is a programmed cell death with distinct morphological characteristics, including cell shrinkage, pyknosis, membrane blebbing, chromatin condensation and formation of cytoplasmic blebs (178, 179). Many diseases are related with apoptosis, such as AIDS (Acquired Immuno-Deficiency Syndrome), neurodegenerative diseases, autoimmune disorders, infectious disease, and cancer (180, 181). All somatic cells including cancer cells need to suppress apoptosis mainly by input of survival and trophic signals to survive (171). Besides, apoptosis also contributes to chemotherapy resistance (182). It’s a potential therapy to target the lesions that suppress apoptosis in tumor cells for cancer apart from inhibiting cancer cell proliferation (183–185). In recent years, postbiotics have been investigated to induce apoptosis in order to treat cancer (172, 173, 186). Postbiotic metabolites produced by six strains of *L. plantarum* induced apoptosis of MCF-7 breast cancer cells (173). Similarly, Kim et al. reported that heat-killed *B. bifidum* MG731, *L. reuteri* MG5346, and *L. rhamnosus* MG5200 induced apoptosis in human gastric cancer MKN1 cell to block tumor cell growth significantly. SCFAs, as one of the intensively investigated postbiotics, were also found to induce apoptosis in cancer treatment (186). SCFAs activated the mitochondrial apoptosis pathway and Fas death receptor apoptosis pathway in the breast cancer cell line 4T1 *in vitro* (186). Combining butyrate together with gemcitabine were found to decrease the percentage of live cells while increasing that of apoptotic cells in human pancreatic cancer cell lines (172). The underlying mechanism of butyrate’s anticarcinogenic effect is to modulate gene expression of key regulators in apoptosis and cell cycle such as cell cycle inhibitor p21 and proapoptotic protein BAK (99, 187–190).

**FIGURE 2:**
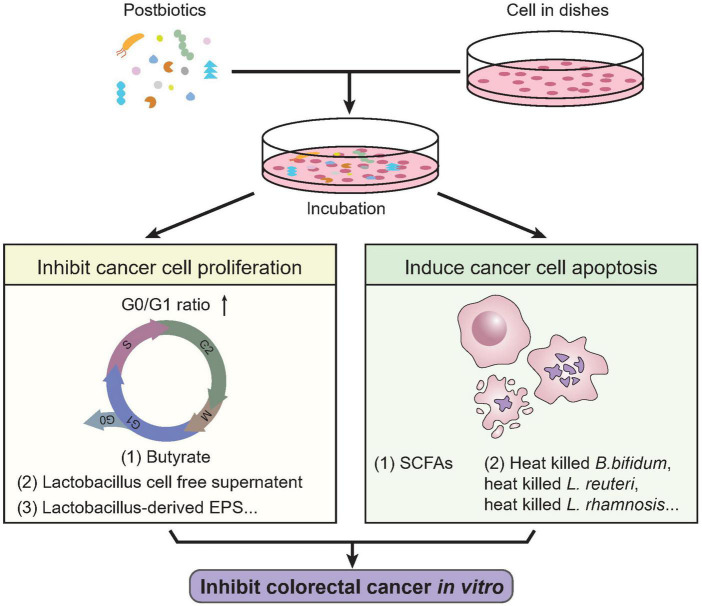
*In vitro* activities of postbiotics against colorectal cancer. Previous studies revealed multiple biological activities of postbiotics against colorectal cancer cells *in vitro*. Certain types of postbiotics like butyrate, *lactobacillus* cell free supernatant, *Lactobacillus* derived EPS, etc., were shown effective in inhibiting colorectal cancer cell proliferation, characterized by cell cycle arrest (increased G0/G1 ratio and decrease in S-phase). On the other hand, SCFAs, heat-killed *B. Bifidum*, *L. reuteri*, *L. rhamnosis*, etc., could induce apoptosis of colorectal cancer cells. These indicate the potential biological activity of postbiotics in treating CRC. SCFAs, short chain fatty acids.

Therefore, it is a potential way to harness postbiotics to treat CRC by inducing colorectal cancer cell apoptosis (183–185). The cultural supernatant of *L. gallinarum* significantly promoted apoptosis in CRC cell lines, HCT116 and LoVo, without impacting the cell growth of normal colonic epithelial cell line (NCM460), showing its specific beneficial potential against CRC (191). Consistently, *Streptococcus thermophilus* could secrete β-Galactosidase promoting CRC cells apoptosis and suppress the growth of CRC xenograft (192). Ma et al. discovered the induction of tumor apoptosis through PI3K/Akt signaling and caspase-3 activation in mice by the increasing production of beneficial metabolites including SCFAs when administrating sitosterol (193). *Clostridium butyricum* can suppress intestinal tumor development by modulating Wnt signaling and gut microbiota, including increasing the abundance of SCFAs-producing bacteria, especially butyrate-producing bacteria, thus suggest their potential efficacy against CRC (194). The *in vitro* activity of postbiotics on colorectal cancer cells is illustrated in Figure 2. On one side, postbiotics can efficiently inhibit colorectal cancer proliferation by disrupting the normal cell cycle of cancer cells; on the other side, postbiotics are effective in inducting cancer cell death through apoptotic pathways.

### 3.2. *In vivo* biological activities of postbiotics

#### 3.2.1. Modulation of gut microbial composition and abundance by postbiotics

The research on the effect of postbiotics on gut microbiota had been emerging in the past few years. It was found that postbiotics could modulate the composition and abundance of gut microbiota by promoting the growth of probiotics in the gastrointestinal tract of both healthy people and patients with different kinds of diseases (32). Terada et al. studied the effects of the consumption of heat-killed *Enterococcus faecalis* EC-12 on microbiota in healthy adults, and found that the levels of *bifidobacteria* and *lactobacilli* were significantly increased, while the abundance of lecithinase-positive *clostridia* including *Clostridium perfringens*, and *Enterobacteriaceae* were significantly decreased (195).

Postbiotics are reported to affect the gut microbiota of individuals with GI disorders, including inflammatory bowel disease (IBD). Sassone-Corsi et al. reported that microcins produced by *Escherichia coli* Nissle 1917 (EcN) could mediate inter and intra-species competition among the *Enterobacteriaceae* in the inflamed gut (196). The two major categories of IBD are Crohn’s disease (CD) and ulcerative colitis (197). In IBD patients, the pattern of gut microbiota dysbiosis is primarily characterized by a reduction in the abundance of bacterial species within the phylum of *Firmicutes* and *Bacteroidetes* and a relative increase of bacterial species belonging to the *Enterobacteriaceae* family, within the Phylum of *Proteobacteria* (198). Sokol et al. found that *Faecalibacterium prausnitzii* supernatant can effectively treat the intestinal microbiota disorders caused by CD and inhibit intestinal inflammation (197). Microbially modified bile acids were correlated with bacterial growth, activation of inflammation, and gut epithelium damage, suggesting an important role as components of intestinal antimicrobial defense (199, 200). It was revealed that cholic acid (primary bile acid) treatment significantly altered the composition of gut microbiota and promoted intestinal carcinogenesis, which is tightly correlated with the transformation of cholic acid to deoxycholic acid *via* bacterial 7α-dehydroxylation reaction, indicating the biological role of deoxycholic acid in gut microbiota homeostasis and CRC progression (201). Additionally, UCDA, which is mentioned earlier, was shown beneficial in improving the outcome of colitis *via* gut miciobiota modulation (202). It was also found that postbiotics could influence behavior in a mouse model of *Citrobacter*-induced colitis. A postbiotic derived from *Limosilactobacillus fermentum* and *Lactobacillus delbrueckii* shortened the small intestine and increased colon crypt depth in the mouse model, showing the protective effect of postbiotics on colitis (203).

The bacteriocin preparation fermented by lactic acid bacteria (LAB), lacidophilin tablets, has been extensively studied (204). Continuous intervention of lacidophilin tablets can reduce harmful bacteria and increase beneficial bacteria and normal bacteria. Lacidophilin tablets could adjust the composition and diversity of intestinal microbiota quickly in antibiotic-associated diarrhea (AAD) mice, and reconstruct the microbial communities which were mainly beneficial bacteria (205, 206). Specifically, lacidophilin-treated mice dramatically increased the abundance of *Lactobacillus* and decreased that of potential pathogens, such as *Bacteroides*, *Parabacteroides*, and *Parasutterella*, to nearly normal level (206). The interaction between postbiotics and anxiety and depressive disorder has also been studied. It showed that ADR-159 treatment, a heat-killed fermentate generated by two *Lactobacillus* strains, led to subtle but distinct changes in murine gut microbiota together with increased sociability and lower baseline corticosterone levels (stress hormone), supporting a beneficial effect on anxiety disorder and depressive disorder (207).

The *in vivo* function of postbiotics in regulating gut microbial composition in the context of CRC has not been fully elucidated. However, accumulating evidence showing the influence of gut microbial composition on CRC progression. Kimoto-Nira et al. investigated influence of long-term consumption of a *Lactococcus lactis* strain G50 on the intestinal flora of the senescence-accelerated mouse, showing that G50 could suppress the intestinal growth of H_2_S-producing bacteria (208). The H_2_S-producing intestinal bacteria include *Salmonella* sp., *Shigella* sp., and *Citrobacter* sp., all of which are associated with CRC (209). Gut microbiota dysbiosis was discovered in CRC patients. *Clostridium butyricum* supernatant was reported to decrease the relative abundance of pathogenic bacteria, such as *Desulfovibrio*, *Odoribacter*, and *Helicobacter*, which are related to CRC (194). Collectively, these results highlight the potential role of postbiotics in the prevention of CRC.

#### 3.2.2. Modulation of gut commensalism by postbiotics

The first line of host defense against both native gut commensals and external pathogens is the intestinal mucosal barrier, which consists of tightly connected epithelial cells and is protected by two host-secreted mucous layers, inner mucus layer and outer mucus layer (210, 211). The intestinal mucus layer was composed of secreted mucin glycoproteins and other substances, which were secreted by goblet cell (212). It is found that mucin can not only provide adhesion sites for intestinal symbiotic bacteria, but can also separate the pathogens from Caco-2 cells (213). However, several intestinal pathogens could secrete enzymes to disrupt the mucous barrier or promote their invasion capability to facilitate their colonization and successful infection (214). Postbiotics could modulate gut commensalism by affecting the intestinal mucosal barrier. Bacterial SCFAs and other metabolites can induce mucus biosynthesis in germ-free mice (215, 216). For example, butyrate-treated human colorectal cells LS174T cells increased mucin protein expression, which enhanced adherence of probiotics *Lactobacillus* and *Bifidobacterium* and eventually inhibited the pathogenic *Escherichia coli* (*E. coli*) attachment (217, 218). Other probiotics supernatant, such as *Lactobacillus rhamnosus* GG culture supernatant, have been proved to promote mucin production to help the gut colonization of symbiotic bacteria and exert a preventive effect on most gut-derived pathogenic infections including *E. coli* K1 (218).

Adhesion is an important step for gut commensalism. If postbiotics provide adhesins such as fimbriae and lectins, they can compete with resident microorganisms for adhesion sites (219, 220). The isolated lectin domains of Llp1 and Llp2 exhibit significant inhibitory activity against biofilm formation of various pathogens, including clinical *Salmonella* species and pathogenic *E. coli* (220). EPS are extracellular polysaccharides attached to the bacterial cell wall which can affect adhesion by shielding cell surface adhesins or acting as ligands (221). It was found that EPS produced by LAB could block the adhesion of harmful bacteria to intestinal epithelial cells by inhibiting binding of adhesion factors to bacterial cell surface (222). Besides, different kinds of EPS could also modulate LAB’s efficiency of adhesion to intestinal epithelial cells *in vitro* (223). In addition to EPS, it was also found that heat-treated *Lactobacillus acidophilus* strain LB could inhibit the adhesion of entero-invasive pathogens to human intestinal Caco-2 cells (224). Thus, postbiotics may exert benefit in treating CRC by modulating gut commensalism through intestinal barrier remodulation (225).

#### 3.2.3. Immunomodulatory activity of postbiotics

Postbiotics can modulate gut pathogenesis not only through competitive inhibition of receptor adhesion but also by changing the intestine barrier function or expression of specific regulatory genes. Tsilingiri et al. first confirmed the anti-inflammatory activity of the *Lactobacillus paracasei* supernatant (SN) after *Salmonella* infection of healthy tissue (226). They showed that the secretion of TNF-α, which is a proinflammatory cytokine, was increased after *Salmonella* infection. Proinflammatory cytokines are positive mediators of inflammation (227). But this effect was eliminated when SN was added together with *Salmonella*, manifesting an inflammatory potential of *Salmonella* (226). Mechanistically, SN can stimulate the epithelium cells resistant to *Salmonella* invasion rather than affecting *Salmonella* proliferation (226). Similarly, acetate was discovered to significantly increase resistance to enterohaemorrhagic *E. coli* O157:H7 infection in a mouse model (228). The could be attributed to the sealing properties of acetate on the intestinal barrier, eventually preventing lethal toxins from entering the general circulation (228). Ramakrishna et al. showed that some probiotics and their metabolites prevent pathogen invasion not only by blocking adherence sites but also by upregulating gene expression of MUC2 and antimicrobial peptides (229). Consistent with this is that Mack et al. showed that part of the beneficial effect of *L. ramnosus* and its supernatant was mediated by induction of mucin genes in intestinal epithelial cells (230).

The gut microbiota dysbiosis could promote the progression of CRC probably through pro-inflammatory responses (231–233). For example, the next generation sequencing analysis revealed that enrichment in *Fusobacterium* could promote inflammation in CRC (234). Inflammatory responses play an important role in different stages including initiation, malignant conversion, invasion, and metastasis of tumor such as CRC development (235). Some anti-inflammatory drugs like aspirin and sulindac were proved to prevent CRC and decrease side effects of inflammatory symptoms, hence bring new insights of CRC prevention and treatment (236, 237). Thus, the immunomodulatory activity of postbiotics can be harnessed in CRC prevention and treatment. For example, bile acids were reported to modulate gastrointestinal inflammation and play a role in CRC prevention (110). Niacin, which is also called nicotinic acid or vitamin B3, can be regarded as another kind of postbiotic because it could also be produced by intestinal microbes and exerts benefits to human health (238). It was reported that niacin can modulate immune responses through G protein-coupled receptor 109a (GPCR109a) to prevent colitis and colon cancer (239, 240). Apart from the anti-inflammation activity, postbiotics can also ameliorate CRC progression by inhibiting the enzymatic activity of pathogenic bacteria and reducing the amount and activity of virulence factors to attenuate gut pathogenesis (241, 242). However, certain microorganisms like *Fusobacterium nucleatum*, *Escherichia coli* NC101 and *Bacteroides fragilis* not only induce inflammation and ROS-mediated genotoxicity but also secrete toxins which induce DNA damage responses to promote CRC (243–245).

#### 3.2.4. Modulation of the gut microbiota-host interactions by postbiotics

Gut microbiota-host interaction is critical for human health. Host inflammatory response is one of the most important signals modulating the homeostasis between gut microbiota and host. It was reported that inflammatory cell infiltration in adipose tissues, liver, and pancreatic islets is associated with an increase in LPS plasma levels, possibly derived from gut bacteria (246, 247). Moreover, the gut microbiota can regulate host inflammation by affecting cytokine secretion as well as the differentiation of inflammatory cell types, demonstrating the dynamic interplay between gut microbiota and the host immune (248, 249).

**FIGURE 3:**
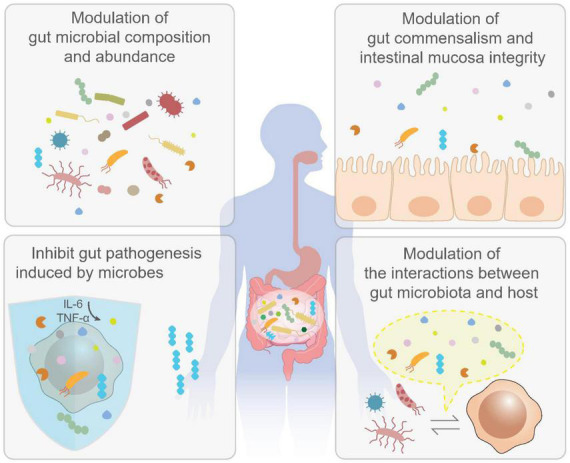
Biological activities of postbiotics *in vivo*. The benefits of postbiotics have also been well-documented by enormous *in vivo* studies. It has been shown that postbiotics are effective in the modulation of gut microbial composition and abundance (upper left); maintaining gut commensalism as well as intestinal mucosa integrity (upper right); inhibit gut pathogenesis induced by gut microbes (lower left) and modulation of interactions between gut microbiota and host and (lower right). These activities may contribute to the prevention and treatment of colorectal cancer (CRC).

For instance, *F. nucleatum* promoted carcinogenesis by stimulating Wnt signaling pathway in CRC (250, 251). And the adhesion molecule FadA from *F. nucleatum* can bind to E cadherin, leading to activation of β-catenin and tumor development (252). *Bacteroides fragilis* was also known as the risk factor of CRC. *Bacteroides fragilis* toxins can bind to specific colonic epithelial receptor to activate Wnt and NF-κB signaling cascades, leading to an increased proinflammatory reactions including T_*H*_17 response and induction of DNA damage response, aggravating the colorectal tumorigenesis (253, 254). All the discussed *in vivo* biological roles of postbiotics in preventing CRC are illustrated in Figure 3. The beneficial roles of postbiotics *in vivo* are achieved *via* the following ways: postbiotics are effective in modulating gut microbial composition and the abundance of certain microbial species, which lead to homeostasis of gut microbiota; postbiotics can also benefit gut commensalism by promoting the integrity of intestinal mucosa tissue; postbiotics can specifically inhibit the invasion of pathogenic microbes in the gut; postbiotics are involved in the interaction between gut microbiota and host cells.

## 4. Molecular mechanisms of postbiotics against colorectal cancer

### 4.1. SCFA

Regarding the molecular mechanism of SCFAs in CRC development, it was shown that butyrate acid could suppress the expression of Neuropilin-1 (NRP-1) by inhibiting the activity of specificity protein 1 (Sp1), which eventually leading to the inhibition of angiogenesis and metastasis of colorectal cancer cells (255). In addition, SCFAs were shown to inhibit CRC cell proliferation by promoting the expression of endocan through extracellular signal-regulated kinases (ERK)/Mitogen-activated protein kinase (MAPK) signals (256). On the other hand, SCFAs induced apoptosis of colorectal cancer cells *via* the upregulation of Bcl-2-associated X protein (Bax) expression (257). G-protein coupled receptors (GPCR) 41/43 and GPCR109a were identified as the binding partners of SCFAs, suggesting the involvement of G protein as well as other downstream signals in mediating SCFAs’ biological functions on the host side (258, 259). Interestingly, it was revealed that deficiency of GPCR43 was associated with reduced abundance of gut commensal bacteria *Bifidobacterium* species and severe gut inflammation, meanwhile, restoration of *Bifidobacterium* sp. in GPCR43 knockout mice attenuated both inflammation and carcinogenesis (260, 261). In contrast, GPCR43 knockout mice exhibited a significantly higher abundance of *Helicobacter hepaticus* and *Prevotellaceae* family strains, both of which are tightly correlated with high intensity of GI inflammation and CRC development (261). These results imply the complicated interplay among SCFAs, gut microbiome, and CRC development. In an independent study, it was found that the lack of GPCR43 was associated with higher incidence of colonic tumor burden in *Apc^min/+^* mice [which is deficient in one allele of the adenomatous polyposis coli (Apc) gene and is highly vulnerable to the development of spontaneous CRC] (262). Mechanistic analysis further revealed that loss of GPCR43 leads to upregulation of cAMP/Protein Kinase A (PKA)/cAMP response element binding protein (CREBP)/histone deacetylase (HDAC) signaling axis, along with upregulated Wnt signaling and the downstream activation of nuclear β-catenin and c-Myc, suggesting the role of G protein-linked epigenetic modification in promoting CRC development (263). In contrast, the involvement of GPCR41 in SCFA-mediated signaling during CRC development has not been fully clarified, except for one study showing that the overexpression of GPCR41 depressed forskolin-stimulated cyclic AMP (cAMP) formation in response to SCFA treatment, similar to the endogenous function of GPCR41 in regulating Gαi/o activity (264). Overexpression of GPCR41 resulted in the inhibition of SCFA-induced histone acetylation, as well as reverse anti-proliferative and apoptotic effects of SCFAs (264). GPCR109a is another known receptor for SCFAs and is predominantly expressed in colon tissues (259). It was reported that GPCR109a is the receptor for butyrate (240, 259, 265). Interestingly, it was found that the expression level of GPCR109a is negatively correlated with colon tumor severity, which was significantly decreased in the normal colon tissue from *Apc^min/+^* mice as compared with that of wild-type littermates, and was nearly absent in the colon tumor tissues of *Apc^min/+^* mice (259). Loss of GPCR109a leads to the increased DNA methylation and upregulated activity of DNA methyltransferases, DNMT1 and DNMT3b (259). GPCR109a activation also resulted in elevated apoptosis through the inhibition of anti-apoptotic genes like B-cell lymphoma 2 (Bcl-2) and significant upregulation of pro-apoptotic effectors including Fas ligand (FasL), Fas associated death domain (FADD), and TNF receptor 1 (TNFR1). In addition, GPCR109a activation also led to the inhibition of nuclear transcription factor κB (NF-κB), which plays a central role in promoting colon inflammation, which may contribute to CRC development (259). Collectively, there are emerging lines of evidence illustrating the molecular mechanism of SCFA in modulating different host responses (240), however, the exact mechanism of SCFA in the context of CRC awaits further investigation.

In addition, recent studies revealed that the overall response of cancer patients following immune checkpoint blockade (ICB) treatments was affected by the composition of gut microbiota (266). Transplantation of fecal microbiota or commensal microbes significantly improved the efficacy of ICBs (267, 268). Notably, oral administration of SCFAs exhibits obvious benefits against cancers suggesting part of the regulatory mechanism of gut microbiota and host immune response (269). As accumulating immune therapeutic strategies are now approved in the clinical treatment of CRC, the role of SCFAs in colorectal cancer immunotherapies would be another interesting topic worth further investigation. Besides, SCFAs also protect the mucosal layer from damage by lowering the level of immune modulators, such as prostaglandins, formed by cyclooxygenase 2 (COX-2). It is reported that the level of COX-2 mRNA is increased in intestinal cancer tissues, and the level of prostaglandin E2, which contributes to inflammation and tumor growth, is also higher than normal tissues (270), while butyrate was found to reduce the expression of COX-2 in tumor tissues to prevent mucosal layer from prostaglandins (270).

### 4.2. EPS

Microorganism-derived components, including EPS, are generally recognized by host innate immune receptors named pattern recognition receptors (PRRs) including toll-like receptors (TLRs), nucleotide oligomerization domain-like receptors (NLRs), Retinoic acid-inducible gene (RIG)-I like receptors and lectin receptors, etc., (271). Notably, C-type lectin receptors (CLRs) can specifically recognize and bind to different kinds of bacteria-derived glycans, which induced receptor dimerization. Downstream signals involve the recruitment of immune tyrosine-based activation motif (ITAM) and activation of spleen tyrosine kinase (SYK), which further activating NF-κB and various immune responses (272). In addition, TLR2 was considered as another receptor of EPS. In 2005, Mazmanian et al. showed that polysaccharide (PSA) from gut commensal bacterium *Bacteroides fragilis* promoted the maturation of the host immune system. The “loss-of-function” mutant of *B. fragilis* in PSA production resulted in systemic T cell deficiencies and Th1/Th2 cell imbalance, indicating the role of PSA in host immune regulation (273). PSA derived from *B. fragilis* activated CD4^+^ T cells and the subsequent predominant Th1 response, which was diminished in TLR2^–/–^ mice (274). TLR-2 pathway promoted the homeostasis of gut immunologic tolerance through sensing the *B. fragilis* derived PSA molecules, leading to activation of Foxp3^+^ regulatory T cells (275). A novel EPS from the biofilm of *Thermus aquaticus* YT-1 was proved to be effective in inducing macrophage activation through TLR-2 signal (276). Collectively, these results revealed the involvement of host innate immune system in sensing and transducing EPS-derived signals as well as their essential role in regulating host immune response.

In addition, accumulating evidence also suggested the immune regulatory activity of EPS on phagocytosis, immune activation, and antigen presentation (277–279). Previous studies demonstrated the capability of host immune system in distinguishing commensal bacteria-derived EPS from that of pathogens (80). It was further revealed that EPS could exhibit opposite immune regulating activities depending on the molecular property. For example, the negatively charged or small EPS can act as stimulators of immune cells, while neutral and large EPS would induce suppressive effects (280, 281). An *in vivo* study showed that EPS from *Bacteroides fragilis* protected animals from *Helicobacter hepaticus-induced* colitis by suppressing the production of IL-17, suggesting the anti-inflammatory activity of EPS and protective role against CRC (282). Recently, Kawaharada et al. identified *epr3* gene in *Lotus japonicus* as the receptor of EPS derived from *Mesorhizobium loti* (283), the molecular structure of which was illustrated later (284). These results provided new insights into the functional mechanism of EPS as well as its role in plant-microbe symbiosis. However, the exact molecular mechanism of EPS in the mammalian system remains unclear. One possible explanation is the huge structural heterogeneity of EPS variants, which makes it difficult to access each host binding partner precisely (80). Overall, with ongoing intensive investigations, the potency of EPS as a promising type of postbiotic candidate in CRC treatment will be elucidated in the future.

### 4.3. Enzymes

To determine the biological role of bacterial enzymes in inhibiting CRC progression, LeBlanc et al. detected the existence of antioxidant enzymes, such as catalase (CAT) or superoxide dismutase (SOD) in the gut and their function in eliminating 2,4,6-Trinitrobenzenesulfonic acid (TNBS)-induced Crohn’s disease in mice, which was believed to be the major trigger of CRC (285). It was indicated that mice received engineered CAT or SOD-producing LAB showed a faster recovery of initial weight loss, increased enzymatic activities in the gut, and less intestinal inflammation compared to that with WT strain or blank controls, suggesting the protective role of antioxidant enzymes in CRC pathogenesis. Carmen et al. further demonstrated that the production of antioxidant enzymes by genetically modified LAB is effective against DMH-induced colorectal cancer in mice model (286). In addition, *Lactobacillus* strains with either the highest catalase activity or the highest dismutase-like activity were assessed for their protective effects on colorectal inflammation (287). It was found that *Lactobacillus* strains with dismutase-like activity were more effective in alleviating intestinal inflammation than strains producing catalase, indicating the crucial role of superoxide anion radical decomposition in this process (287). Collectively, the biological activity of bacterial enzymes in alleviating colorectal inflammation or preventing CRC has been uncovered, while the underlying mechanism remains unclear.

### 4.4. Other postbiotics

The relationship between bile acid transportation and gut microbial metabolism, as well as the implications on CRC development, has received more and more attention in recent years. Numerous studies have demonstrated the carcinogenic activity of bile acids, especially the secondary bile acids, in the development of colorectal cancer (288). It was revealed that bile acids disrupt colonic epithelial cells by inducing reactive oxygen species (ROS) production, genome destabilization as well as other effects, which eventually lead to colon tumorigenesis (289–291). Mechanistic studies further showed the involvement of MAPK, phosphoinositide 3-kinase (PI3K)/Akt and NF-κB in this process (292, 293). Despite this, other studies revealed the pro-apoptotic effect of the secondary bile acid deoxycholate acid, which is produced by gut microbiota, on colorectal cancer, as treatment with as low as 0.5 mM deoxycholate acid was able to induce apoptosis of colorectal cancer cells, which was comparable to the activity of SCFAs (294). However, the molecular mechanism remains unclear. A recent study illustrated different functions of primary and secondary bile acids in regulating host immune response against liver cancer (295). Specifically, primary bile acids produced by host metabolism increased CXCL16 expression, which could recognize and bind to CXCR6 expressed on natural killer T (NKT) cells. This interaction eventually led to hepatic tumor infiltration and tumor targeting of NKT cells. In contrast, secondary bile acids processed by gram-positive bacteria in the gut exhibited an opposite effect (295). Taken together, the exact role or molecular mechanism of bile acids, especially the microbial derived secondary bile acids in CRC development remain controversial, which relies on future studies to be better addressed.

**FIGURE 4:**
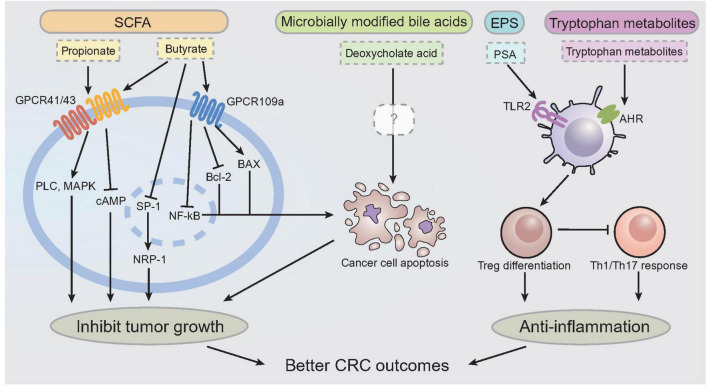
Molecular mechanism of postbiotics on colorectal cancer prevention and treatment. Working mechanisms of postbiotics against CRC. It has been shown that propionate and butyrate can bind to GPCR41/43 to promote the activation of PLC, MAPK, and signals intracellularly to inhibit tumor cell growth. Butyrate also binds to GPCR109a, which inhibits downstream intracellular signals including SP-1, NRP-1, to inhibit tumor cell growth, and also promote apoptosis *via* BAX and inhibition of Bcl-2, NF-kB activity. Deoxycholate acid, a type of microbially modified bile acids, can promote cancer cell apoptosis through unidentified mechanism. PSA interacts with TLR2 to promote the activation of DCs, which lead to the upregulation of T regulatory cells in GI environment. Tryptophan metabolites interact with AHR to exert the anti-inflammatory activity as well. GPCR, G-protein couple receptor; PLC, phospholipase C; MAPK, mitogen-activated protein kinase; cAMP, cyclic AMP; SP-1, Specific protein 1; NRP-1, Neuropilin-1; PSA, polysaccharide; TLR, toll like receptor; DC, dendritic cell; AHR, aryl hydrocarbon receptor; CRC, colorectal cancer.

For the working mechanism of tryptophan metabolites, it was found that indole metabolites and kynurenine can interact with aryl hydrocarbon receptor (AHR) to induce T regulatory cells differentiation, confine Th17 and Th1 responses and produce anti-inflammatory mediators (296, 297); kynurenine reduces tumor-infiltrating CD8^+^ T cells and mediates immune evasion of tumor cells (298), while indole metabolites alleviate colitis and protect against colorectal cancer (299, 300). Serotonin (5-HT) was also found to be critical in regulating GI inflammation, while the exact activity remains controversial. Evidence proved the anti-inflammatory role of serotonin in colitis, while others revealed the enhanced effect of serotonin in colorectal cancer progression (301). The interaction between tryptophan metabolites and host AHR is essential for regulating colon barrier function and inducing regulatory T cell differentiation (302). The association between fecal tryptophan metabolites and gut barrier functions was further validated by a clinical study, implying the involvement of tryptophan metabolism in the development of CRC (136). Figure 4 illustrated the presently identified working mechanisms of different postbiotics that are beneficial for CRC protection. Briefly, SCFAs (e.g., propionate and butyrate) can modulate cancer cell growth and promoting apoptosis of cancer cells *via* GPCR41/43/109a. Microbially derived secondary bile acid, notably deoxycholate acid, can efficiently induce cancer cell death *via* apoptotic pathway, which mimics SCFAs. EPS, notably PSA, can modulate Treg cell differentiation and activation through TLR2 sensing. Tryptophan metabolites are also effective in DC induced Treg differentiation *via* AHR sensing, which suppresses pro-inflammatory Th1/Th17 response. All these activities are implied in preventing CRC progression.

## 5. Clinical potency of postbiotics in CRC

### 5.1. Description of analysis

To obtain an complete picture of our current understanding regarding the clinical benefits of postbiotics, especially in cancer treatment, we went through multiple online databases [PubMed, EMBASE, China National Knowledge Infrastructure (CNKI), Wanfang, etc.] to collect clinical studies published on or before the date of September 18, 2022 using following keywords: “postbiotics,” “Heat-killed bacterial cells,” “Cell-free supernatant,” “Teichoic acids,” “Exopolysaccharides,” “Short chain fatty acids,” “Enzyme,” “Yeast cell wall,” “Bile acid,” “Tryptophan,” “Bacteriocins,” “Vitamin,” “Neurotransmitter substance,” “Microbiota,” “therapy,” and “cancer,” without any other restrictions.

A total of 538 literatures were systematically reviewed, and only a few original clinical studies investigating the effects of postbiotics on the outcome of cancer patients were selected for further analysis, which are listed in Table 2 and explicitly described as below (Table 2).

**TABLE 2:** Characteristics of selected clinical studies related to postbiotics.

Year	Country	Participants T vs. C	Sex ratio T(M:F) vs. C(M:F)	Age	Disease	Intervention (Postbiotics)	Primary endpoint	Secondary endpoint	Randomization	Blinding	References
2021	USA	103 vs. 41	/	63.6 (55.6–73.0) vs. 59.4 (49.5–67.8)	melanoma	TLPLDC Vaccine (Yeast cell wall)	2 years DFS	36 months DFS and OS	Y	double-blind	(303)
2000	UK	14 vs. 15	(11:3) vs. (9:6)	61 (33–71) vs. 59 (40–70)	NSCLC and mesothelioma	SRL172	Median survival time	RR	Y	NI	(304)
2007	UK	18 vs. 18	(17:1) vs. (12:6)	60 (45–58) vs. 55 (48–69)	renal cell carcinoma	SRL172	TTP	Toxicity, RR and OS	Y	NI	(306)
2001	UK	14 vs. 14	(9:5) vs. (6:8)	61.5 (46–73) vs. 60.5 (48–75)	Small Cell Lung Cancer (SCLC)	SRL172	Median Survival time	1 year survival	Y	NI	(305)

Summary of current clinical studies based on the criteria described in the main text. USA, united states of america; UK, united kingdom; T, treatment; C, control; M, male; F, female; NSCLC, non-small cell lung cancer; SLCL, small cell lung cancer; TLPLDC, tumor lysate, particle-loaded, dendritic cell; SRL172, heat-killed Mycobacterium vaccae (Heat-killed microorganism cells); DFS, disease-free survival; TTP, time to progress; OS, overall survival; RR, response rate; Y, Yes; NI, No Information.

### 5.2. Major findings and interpretation

Among the selected studies, Vreeland et al. launched a randomized, double-blind, placebo-controlled trial in order to evaluate the clinical efficacy of “Tumor Lysate, Particle-Loaded, Dendritic Cell (TLPLDC)” vaccine as adjutant therapy to advanced-stage melanoma, which declared that patients enrolled in TLPLDC group had longer 24 months disease-free survival (62.9 vs. 34.8%, *p* = 0.041) (303). In addition, O’Brien et al. had demonstrated that traditional chemotherapy combined with heat-killed Mycobacterium vaccae SRL172 improved the response rate, median survival and 1 year survival by randomized trials in non-small cell lung cancer (NSCLC) and mesothelioma. Moreover, they also found that SRL172 could improve the situation of sleep (*p* = 0.08) and appetite (*p* = 0.01) of the patients (304). Assersohn et al. had applied traditional chemotherapy combined with heat-killed Mycobacterium vaccae SRL172 to Small Cell Lung Cancer (SCLC), which improved the median survival of the patients (305). Patel et al. suggested that patients with renal cell carcinoma treated with SRL172 plus IL-2 reduced adverse events in comparison to those treated with IL-2 alone (*p* < 0.001) (306). Unfortunately, none of those studies directly accessed the therapeutic effect of postbiotics on CRC patients.

Overall, the clinical effects of postbiotics in cancer prevention/treatment, in particular CRC, are still lacking as few studies have been drown by far. Additional evidence are still required to demonstrate the potential clinical effects of postbiotics in CRC prevention and treatment, which relied on well-designed randomized placebo-controlled clinical intervention trials.

## 6. “Pre-Pro-Post”: Comparison of different strategies in CRC treatment

### 6.1. Conceptual difference of prebiotics, probiotics, and postbiotics

With growing interest in uncovering the relationship between gut microbiota and human health in recent decades, different gut microbes are identified to be either beneficial or harmful in the pathogenesis of human diseases. Microbes that could promote human health or prevent disease are termed probiotics and emerged as novel strategies in clinical treatments (307). In 2014, the ISAPP defined probiotics as “live microorganisms that, when administered in adequate amounts, confer a health benefit on the host” (31). Dietary interventions, on the other hand, are more convenient in modulating gut microbiota without causing noticeable side effects. Effective components in such diets are called prebiotics and were also widely accepted as important supplementations by clinicians, researchers and consumers (308). In 2017, ISAPP further defined prebiotics as “a substrate that is selectively utilized by host microorganisms conferring a health benefit” (30). With the accumulation of research on probiotics, prebiotics, and microorganisms, it was found that inanimate microorganisms and their metabolites also behave beneficial activities. The first consensus definition of postbiotics was proposed in 2021, when ISAPP defined postbiotics as “preparation of inanimate microorganisms and/or their components that confers health benefits on the host” (31).

### 6.2. Efficacy/side-effects of different strategies on CRC treatments

Fecal microbiota transplantation (FMT), an emerging approach developed and used by clinicians recently, also achieved promising effects in treating multiple kinds of diseases including CRC (309, 310). While some studies provided mechanistic insights into how probiotic may confer benefits, they were largely performed in *in vitro* models. The clinical evidence for the efficacy of current probiotics or prebiotics as in medical use is still insufficient and inconsistent (311). In addition, many factors may affect the efficiency of exogeneous microbial implantation, including the colonization resistance by residing commensal species (312, 313). Individual response to probiotic colonization or dietary intervention also varies that further dampens the efficacy of “one strain fits all” strategy (314). FMT also introduces uncertainties from donor and fecal materials, which leads to unpredictable outcome in clinic regarding to efficiency or safety (315). Refined FMT strategy with defined consortium of cultured bacterial species seems to be able to overcome these limitations (316). Another major limitation of prebiotic or probiotic administration is the lack of clarity of defined functional mechanisms. Although previous studies have deciphered the molecular interplays among dietary intervention/probiotic administration, gut microbiota, and host response, a large proportion of studies are still observational or correlational. Thus, a comprehensive understanding of the causal interactions is urgent. Furthermore, the safety concerns of probiotic supplementation and FMT are also raised in pre-clinical studies (317).

In contrast, postbiotic administration is outperformed in both efficacy and safety to meet clinical requirements (28). As composed of derivatives from gut microbes, postbiotics refers to “chemicals” rather than “organisms.” With their natural existence in human gut, the safety issue in clinical use is feasible to be addressed through well-designed preclinical and clinical trials. Several studies found that probiotics could cause infectious risks. For example, Wagner et al. colonized athymic mice with human isolates of *Lactobacillus reuteri, Lactobacillus acidophilus, Bifidobacterium animalis*, or *Lactobacillus rhamnosus* GG (LGG). The result showed that colonization with the probiotics *L. reuteri* and LGG led to death in some athymic neonatal mice. This finding suggests that due to the nature of immune deficiency in neonates, it may put them at particularly high risk of sepsis when administrating probiotics (318). Besides, improper administration of probiotics may increase the risk of the spreading of drug resistant strains. For instance, *Lactobacillus* (such as *Lactobacillus plantarum*), which have plenty resistance factors, may become the transmission channel of antibiotic resistance genes (319). In terms of stability, researchers found that postbiotics are supposed to be more stable than the living bacteria they are derived from. That is, postbiotics are more suitable for long term use than probiotics. Venema et al. reported that peptides with antimicrobial properties, namely bacilysin and chlorotetaine, produced by *Bacillus* sp. strain CS93 are water soluble and active over a wide pH range, which could allow their application in a wide variety of food products (320, 321). Furthermore, for postbiotics, the characterization of molecular property, as well as elucidation of underlying mechanisms, would be much more straightforward through current approaches (28). In Table 3, we summarized the features, advantages and disadvantages of different strategies in treating CRC.

**TABLE 3:** Comparison of different strategies in treating colorectal cancer (CRC).

	Efficacy	Safety	Other considerations	References
Dietary intervention	Varied among individuals;	High	Difficult to access the effective components Difficult for therapy standardization	(325)
Prebiotics administration	Varied among individuals	Moderate (compared with probiotic administration)	Insufficient evidence of the biological role in preventing/treating CRC Lack of identified molecular mechanisms	(326, 327)
Fecal material transplantation (FMT)	Varied among individuals	Low (high risk of causing infectious and other diseases)	Delivery methods need to be improved Standards for clinical use are lacking	(310)
Probiotics administration	Varied among individuals Efficiency of implantation is affected by various factors	Low (high risk of infection and spreading of drug resistance microbes)	Insufficient clinical evidence Molecular mechanisms are lacking	(328, 329)
Postbiotics administration	High and stable	High (postbiotics are naturally existed molecules in human gut)	More types of postbiotics need to be identified Additional research needs to be done to address the clinical benefits of postbiotics in CRC prevention/treatment	(36, 330)

Summarization of the characteristics of different types of microbiota-related strategies against CRC, including the efficacy, safety as well as other issues need to be considered for clinical applications.

## 7. Conclusion and future perspectives

Over the last decades, the potency of postbiotics in treating various diseases has been uncovered by accumulating evidence. Compared with other microbiota manipulating strategies like dietary intervention, probiotic administration, or fecal material transplantation (FMT), postbiotic administration is superior in both safety and efficacy, which benefits from the clarification of their molecular identity and functional mechanism. However, there are still challenges in microbiome targeted therapy. First, only a limited categories of postbiotics are known, mainly restricted by the available techniques. Integrative methods including high-throughput multi-omics analysis, computational processing, large-scale screening and animal models are considered as the future direction for identification of new bioactive molecules from gut microbiota (322). Second, human microbiome may have unexpected interaction with supplemental metabolites, which may cause dysbiosis or transformation of metabolites into inactive or even toxic states. In addition, artificial upregulation of certain metabolites in human gut may affect the homeostasis of the endogenous metabolic feedback loop, which will cause the acquired deficiency in the production of metabolites by the human body (323). More importantly, low levels of a given metabolite in fecal sample do not precisely reflect its physiological roles in the gut where it exerts benefits. Multiple sampling sites would be an improvement in overcoming this limitation (324). Overall, it is necessary to systemically identify the existence of new bioactive compounds from gut microbes in the human body as well as their interaction between metabolite and resident microbiome prior to their utilization. Finally, commercialization of postbiotics should be controlled in a cost-effective way, which cannot be achieved by lab-based approaches and largely rely on the optimization of synthesis strategies by industries (35).

To systemically analyze the effects of different postbiotics conducted by different studies, appropriate classification and name of each kind of postbiotic is recommended in the future. Since the characterization of microbial source is a pre-requirement for a given postbiotic to be determined, it is an applicable way to name postbiotics by their microbial source and molecular class/characteristics (e.g., *Bifidobacterium* sp. derived cell free supernatant, or *Lactobacillus* sp. derived exopolysaccharides). Meanwhile, for those substances that can be found in multiple microbial species and the molecular structures have been identified (e.g., butyrate acid, propionate acid, etc.), the chemical name can be used instead for clearance.

Taken together, postbiotic administration is promising in preventing and treating CRC through the modulation of gut microbiota, which has been validated by a large number of preclinical studies. However, the clinical evidence of postbiotics in protecting against CRC is still insufficient. Given the fact that dietary intervention is critical in modulating gut microbiota and are considered as the major factor in CRC development, it is reasonable to expect a positive clinical outcome by precision nutrition. Therefore, large-scale, and double-blind clinical trials will be required in the future to promote the clinical utilization of postbiotics in CRC patients.

## Author contributions

N-NL conceived and designed the manuscript. DS, XW, and YM wrote the manuscript and drafted the figures and tables. N-NL and HW revised the manuscript. All authors have read and approved the final manuscript.
